# The Impact of the COVID-19 Pandemic on Nursing Care: A Cross-Sectional Survey-Based Study

**DOI:** 10.3390/jpm11100945

**Published:** 2021-09-23

**Authors:** Marco Clari, Michela Luciani, Alessio Conti, Veronica Sciannameo, Paola Berchialla, Paola Di Giulio, Sara Campagna, Valerio Dimonte

**Affiliations:** 1 Department of Public Health and Pediatrics, University of Torino, 10126 Turin, Italy; marco.clari@unito.it (M.C.); michela.luciani@unito.it (M.L.); paola.digiulio@unito.it (P.D.G.); sara.campagna@unito.it (S.C.); valerio.dimonte@unito.it (V.D.); 2 Department of Cardiac, Vascular Sciences and Public Health, University of Padova, 35128 Padua, Italy; veronica.sciannameo@unito.it; 3 Department of Clinical and Biological Sciences, University of Torino, 10043 Orbassano, Italy; paola.berchialla@unito.it

**Keywords:** COVID-19, nursing care, patient care planning, quality of health care, personalized care, conditional inference trees

## Abstract

The COVID-19 pandemic has had a severe impact on nursing care. This cross-sectional survey-based study compared aspects of nursing care and nurses’ satisfaction with care provided before and during the first wave of the COVID-19 pandemic. A total of 936 registered nurses (RNs) rated the frequency with which they performed fundamental care, nursing techniques, patient education, symptom management, and nurse–patient relationships before and during the pandemic. A recursive partitioning for ordered multivariate response in a conditional inference framework approach was applied. More frequent fundamental cares were associated with their frequency before the pandemic (*p* < 0.001), caring for COVID-19 patients (*p* < 0.001), and workplace reassignment (*p* = 0.004). Caring for COVID-19 patients (*p* < 0.001), workplace reassignment (*p* = 0.030), and caring for ≤7.4 COVID-19 patients (*p* = 0.014) increased nursing techniques. RNs in high-intensity COVID-19 units (*p* = 0.002) who educated patients before the pandemic, stopped this task. RNs caring for COVID-19 patients reported increased symptom management (*p* < 0.001), as did RNs caring for more non-COVID-19 patients (*p* = 0.037). Less frequent nurse–patient relationships before the pandemic and working in high-intensity COVID-19 units decreased nurse–patient relationships (*p* = 0.002). Despite enormous challenges, nurses continued to provide a high level of care. Ensuring the appropriate deployment and education of nurses is crucial to personalize care and to maintain nurses’ satisfaction with the care provided.

## 1. Introduction

From 31 December 2019, when the World Health Organization’s China Office reported a case of pneumonia of unknown etiology in Wuhan, the Coronavirus Disease 2019 (COVID-19) started to spread across the globe [1]; on 11 March 2020, COVID-19 was declared a pandemic [2]. Italy was one of the first countries outside of China to report cases [1]. As of July 2021, Italy has reported more than 4,200,000 confirmed COVID-19 cases and more than 128,000 COVID-19 deaths [3]. The COVID-19 pandemic has had a severe impact on healthcare systems around the world, affecting the availability of beds in hospitals and intensive care units [4]. The second and following waves of the COVID-19 pandemic are still challenging healthcare systems and professionals [5].

Nurses have been recognized as fundamental actors in public health crises and have played a major role in the COVID-19 pandemic; however, the pandemic has had a severe impact on nursing care. This is due to the challenges associated with the preparedness and response to emergencies shown by several healthcare systems in different care settings [6,7]. In the face of a heavy workload, nurses have had to wear personal protective equipment (PPE) [4,8] and have been confronted with a lack of PPE [9,10], staff [10,11], and other resources [9,12], all of which have led to decreased mental health and well-being [13,14], occupational satisfaction [15], and high infection rates among nurses [16]. Nurses also reported being reassigned due to changes in human resource allocations, having to quickly learn new skills and competencies, having to work with newly-graduated nurses, and difficulties in communicating with patients and their families due to PPE and isolation. All of the factors mentioned above could have affected personalized care, an essential aspect of nursing, during the COVID-19 pandemic [17]. The concept of personalized health care in nursing is influenced by the care environment and the ability of general nursing care to meet a patient’s needs, which were inevitably affected by the emergency, thereby potentially impacting clinical outcomes and satisfaction with care.

However, to the best of our knowledge, no rigorous study exists on how the pandemic has impacted nursing care. In particular, no studies have examined differences in the care provided by nurses who cared for COVID-19 and non-COVID-19 patients, or between those who were reassigned due to the pandemic and those who continued to work in their unit. Moreover, there is still a limited understanding of the factors associated with nurses’ satisfaction with the care provided during the pandemic. This information could help decision makers ensure that nurses in a given unit have the appropriate education, skill mix, and patient-to-nurse ratio, thereby improving clinical practice and care personalization during this pandemic [5] and future health emergencies. Hence, this study aimed to identify changes in nursing care by comparing aspects of nursing care and satisfaction with care provided before and during the first wave of the COVID-19 pandemic, examining differences between nurses who cared for COVID-19 and non-COVID-19 patients, and between those who were reassigned and those who continued to work in their unit.

## 2. Materials and Methods

### 2.1. Study Design and Participants

This cross-sectional study included registered nurses (RNs) in Italy who delivered nursing care in the 3 months before study enrollment. No restrictions regarding the type of patient nor the setting were applied. 

### 2.2. Procedures

RNs were invited to complete an online questionnaire, which was available between 12 May and 31 July 2020. Invitations were disseminated through ads on social media (Facebook, Twitter, Instagram), informational links on the websites of the Nursing Councils, and through texts and e-mails sent directly to RNs, using contact lists obtained from nursing schools in each of the Italian regions. Every 2 weeks, ads and informational links were reposted, and texts and e-mails were resent to RNs. The response rate (RR) was calculated as the RR2 [18], i.e., the sum of complete (I) and partial (P) questionnaires divided by the sum of complete, partial, non-questionnaires (NC, defined as respondents who logged on to the questionnaire but did not complete any item), and other (O, defined as respondents who could not fit in any of previous classifications; in this study, this category was not present): RR2 = (I + P)/((I + P) + (NC + O)). For this study, the RR2 was 81.4%.

### 2.3. Instruments

The online questionnaire was composed of six sections that covered: (I) changes in nursing care due to the COVID-19 pandemic; (II) changes in work organization; (III) ethics choices; (IV) most challenging case; (V) additional education needed to care for COVID-19 patients; (VI) socio-demographic characteristics. In the present analysis, only sections I, II, and VI were considered.

In section I, RNs reported the frequency with which they carried out the following tasks: fundamental care (i.e., personal hygiene, elimination, nutrition, mobility) [19], nursing techniques (i.e., respiratory support, vascular access, device positioning and management), patient education (i.e., respiratory exercises, medication, education), symptom management (pain, dyspnea, fatigue), and nurse–patient relationships (i.e., personal interactions with patients). These frequencies were reported for two time periods: before the COVID-19 pandemic and during what RNs perceived to be the worst week of the pandemic, using a 5-point Likert-type scale (1 = never; 2 = rarely; 3 = sometimes; 4 = often; 5 = most of the time). For each time period, RNs were also asked to rate their overall satisfaction with the care they provided (1 = very poor; 2 = poor; 3 = fair; 4 = good; 5 = excellent).

In section II, RNs reported the number of patients they cared for before the COVID-19 pandemic, if that number increased, remained stable, or decreased during the pandemic, if they were reassigned to another unit, and, if so, how many times. RNs were also asked if they thought there was enough time to prepare for the pandemic in terms of work organization, education/training, and their personal lives, with responses given on 5-point Likert-type scale (1 = not at all; 2 = not really; 3 = neutral; 4 = somewhat; 5 = very much). Finally, RNs were asked to report the number of patients they personally cared for during their last shift, and those caring for COVID-19 patients were also asked to report the number of patients who required no respiratory support, high-flow oxygen, non-invasive ventilation, and mechanical ventilation. The number of patients was then weighted based on the respiratory support provided, with a higher coefficient for patients with mechanical ventilation and a lower coefficient for those with no support.

### 2.4. Analysis

Continuous variables were described using medians and interquartile ranges (IQRs), or means and standard deviations (SDs). The Mann–Whitney U test was performed to evaluate differences in quantitative variables, and Chi-square or Fisher’s exact test was used for categorical variables as appropriate.

A recursive partitioning for ordered multivariate response in a conditional inference framework approach was applied. Conditional inference trees were constructed to identify the pattern of work organization, nurse education/training, and personal lives associated to the different levels of fundamental care, nursing techniques, patient education, symptom management, and nurse–patient relationships in post COVID-19.

Independent variables were selected and split through multiplicity-adjusted *p*-values following the Bonferroni’s criterion. The split of variables determines a set of rules associated to different values of the dependent variable. In more detail, for each variable, the conditional regression tree determines the optimal split and a partitioning is performed, selecting the input variable with the highest multiplicity-adjusted *p*-value. Then, a binary split is performed on the selected input variable, and this process is recursively performed until a stopping criterion is reached. The stopping criterion was based on significant results, i.e., splitting continues until the minimum of the adjusted *p*-values is less than a pre-specified level of significance (0.05) or otherwise stops [20].

In our analysis, a conditional inference tree was constructed for each nursing task as dependent variables (fundamental care, nursing techniques, patient education, symptom management, and nurse–patient relationships). Independent variables included in the trees were: values from before the pandemic for investigated nursing tasks, caring for COVID-19 patients, gender, age, education, geographic area, work experience, working unit, workplace reassignment, preparedness (in terms of work organization, education/training, and personal lives), decrease/increase in number of patients, number of non-COVID-19 patients, a weighted sum of COVID-19 patients with different respiratory support, and type of contract.

We also performed sensitivity analyses using multivariate logistic regression. Likert scores for the investigated nursing tasks before and during the COVID-19 pandemic were dichotomized into never/rarely/sometimes (0) and often/most of the time (1), and odds ratios and 95% confidence intervals were computed. Missing data were deleted listwise. Analyses were performed using R version 3.6.1 [21].

### 2.5. Ethics

RNs were informed about the study before accessing the online questionnaire, and consent was obtained before they began the questionnaire. RNs were not compensated for their role in the study, and participation was voluntary. All data were collected anonymously, and respondents could leave the questionnaire at any time. The study was approved by the University of Torino Ethics Committee (Approval no. 279061–01/07/2020) and conducted following the Declaration of Helsinki guidelines.

## 3. Results

A total of 936 RNs completed the online questionnaire (68.2% female); the median age in the sample was 39 years (IQR 30–49), and 40.7% of RNs had a bachelor’s degree (Table 1).

Most RNs worked in Northern Italy during the pandemic (86.5%). The median work experience was 13 years (IQR 5–25). Most RNs worked in a hospital setting (67.8%), 77.1% cared for COVID-19 patients, 28% worked in a dedicated COVID-19 unit, and 35.9% were reassigned following reorganizations to increase beds for COVID-19 patients. Almost half of RNs reported that they cared for fewer patients during the pandemic (45.8%): the median patient-to-nurse ratio among RNs caring for non-COVID-19 patients was 8 (IQR 3–15), compared to 2.4 (IQR 1–5) among those caring for COVID-19 patients. RNs caring for COVID-19 patients were significantly younger and had less work experience. Furthermore, there was a higher number of male RNs among those caring for COVID-19 patients. Lastly, in areas of high COVID-19 prevalence (i.e., Northern Italy and the Lombardy Region) more RNs were reassigned to COVID-19 hospital units. RNs reported there was little time to prepare for the pandemic in terms of work organization (median 2 (rarely), IQR 1–3), education/training (median 2, IQR 1–2), and their personal lives (median 1 (no time), IQR 1–2) (Table 1).

The highest number of reassigned RNs was observed in the Lombardy Region (19.0% versus 10.1%; *p* = 0.003). Reassigned RNs (*p* = 0.042; median 38, IQR 29–48 versus median 40, IQR 30–50 among those not reassigned) felt poorly prepared in terms of work organization (*p* = 0.004; median 1, IQR 1–2 versus median 2, IQR 1–3) and their personal lives (*p* = 0.009; median 1, IQR 1–2 versus median 1, IQR 1–2) (Table 2). 

Reassigned nurses reported caring for a higher number of patients (*p* < 0.001), and they comprised a higher number of self-employed (9.2% versus 3.8% among those not reassigned; *p* = 0.028) and temporary contract workers (public temporary 3.3% versus 2.6%; private temporary 3.9 versus 2.9). RNs were mostly reassigned from medium–low-intensity facilities, and especially from community care to tertiary care hospitals (*p* = 0.003); RNs reassigned to COVID-19 units were most often transferred from medium–low-intensity facilities to tertiary care hospitals (*p* < 0.001). Conversely, RNs who were not reassigned and did not care for COVID-19 patients were significantly older (*p* < 0.001; median 45.5, IQR 36–51 versus median 37.5, IQR 30–48 among reassigned RNs who did care for COVID-19 patients) and had more work experience (*p* < 0.001; median 20, IQR 10–30 versus median 11, IQR 4–23) (Table 2).

### Nursing Tasks

**Figure 1:**
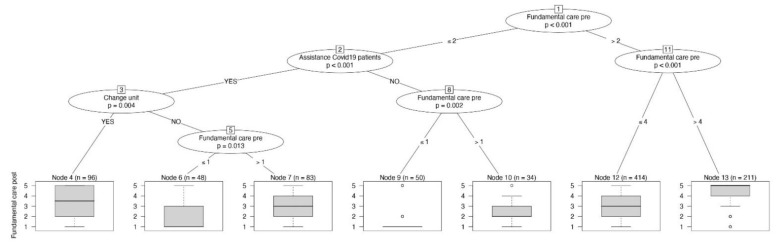
The conditional inference tree with the frequency of fundamental care tasks as dependent variable, reported at the bottom of the figure as boxplot.

The frequency of fundamental care tasks before the pandemic was associated with the frequency during the pandemic (*p* < 0.001; before: median 3, IQR 2–4; during: median 3, IQR 2–5). In Figure 1, the conditional inference tree with the frequency of fundamental care tasks as a dependent variable, reported at the bottom of the figure as a boxplot, is represented. At the top of Figure 1, we can see that the first split of the decision tree was performed based on the frequency of fundamental care registered in the pre-pandemic period (node 1). RNs who frequently performed fundamental care before the pandemic continued this practice (*p* < 0.001), as we can see in the rightmost branch of the tree. Furthermore, among those who declared to not usually perform fundamental care before the pandemic, caring for COVID-19 patients (*p* < 0.001) or being reassigned (*p* = 0.004) significantly increased the frequency of fundamental care during the COVID-19 pandemic, as expressed in the boxplot on the Likert scale, reported in the bottom of the Figure 1, left part.

**Figure 2:**
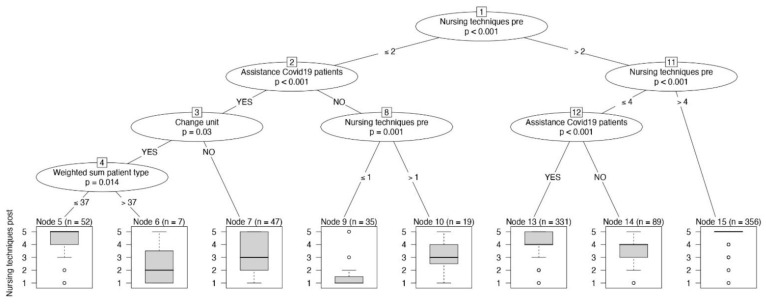
The conditional inference tree with the frequency of nursing techniques as dependent variable, reported at the bottom of the figure as boxplot.

In Figure 2, the conditional inference tree about fundamental care is represented. RNs caring for COVID-19 patients who frequently performed nursing techniques before the pandemic continued this practice (*p* < 0.001; before: median 4, IQR 3–5; during: median 5, IQR 4–5), as we can see by following the rightmost branch. RNs who cared for COVID-19 patients (*p* < 0.001), those who were reassigned (*p* = 0.030), and those who assisted ≤7.4 patients (*p* = 0.014) significantly increased the frequency of nursing techniques, with a median Likert scale of 5, as shown in the leftmost boxplot (Figure 2). 

**Figure 3:**
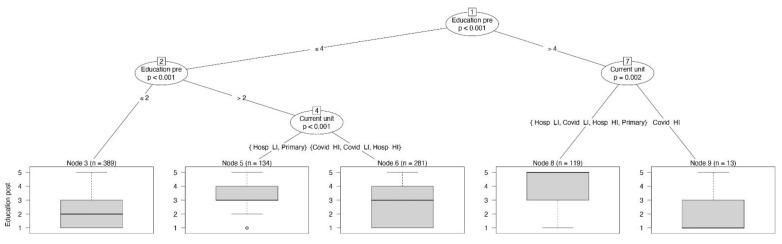
The conditional inference tree with the frequency of patient education as dependent variable, reported at the bottom of the figure as boxplot.

RNs who performed patient education often before the pandemic and worked in high-intensity COVID-19 units (*p* = 0.002) stopped performing this task, as shown in the rightmost branch in Figure 3, while those in other settings continued their usual practice (*p* < 0.001; before: median 3, IQR 2–4; during: median 3, IQR 1–4).

**Figure 4:**
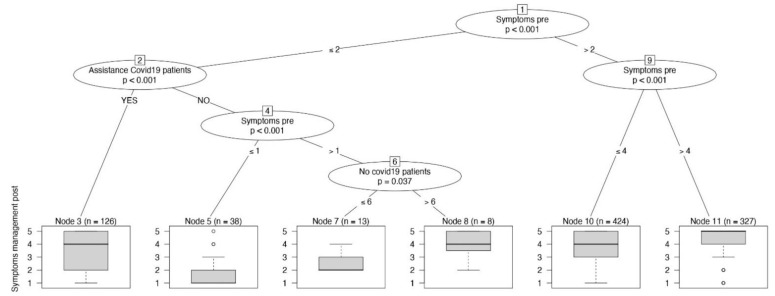
The conditional inference tree with the frequency of symptom management as dependent variable, reported at the bottom of the figure as boxplot.

In Figure 4, the decision tree with symptom management as the outcome variable is reported. The frequency of symptom management during the COVID-19 pandemic was similar to that before the pandemic (*p* < 0.001; before: median 4, IQR 3–5; during: median 4, IQR 3–5), but it increased for RNs who cared for COVID-19 patients (*p* < 0.001), as shown in the leftmost branch. Caring for a higher number of non-COVID-19 patients (>6) increased the frequency of symptom management (*p* = 0.037), as reported in the eighth node, with a median Likert scale of 4.

**Figure 5:**
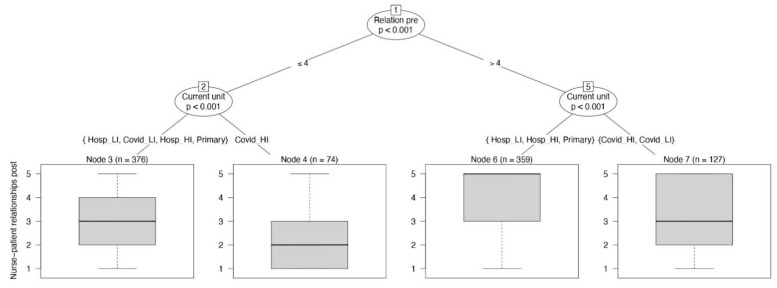
The conditional inference tree with the frequency of nurse–patient relationships as dependent variable, reported at the bottom of the figure as boxplot.

Nurse–patient relationships before the pandemic were associated with those during the pandemic (*p* < 0.001; before: median 5, IQR 4–5; during: median 4, IQR 2–5), but nurses working in high-intensity COVID-19 units who reported frequent nurse–patient relationships before the pandemic had no chance to relate with patients during the pandemic (*p* = 0.002), as shown in the rightmost branch of the conditional decision tree reported in Figure 5. 

**Figure 6:**
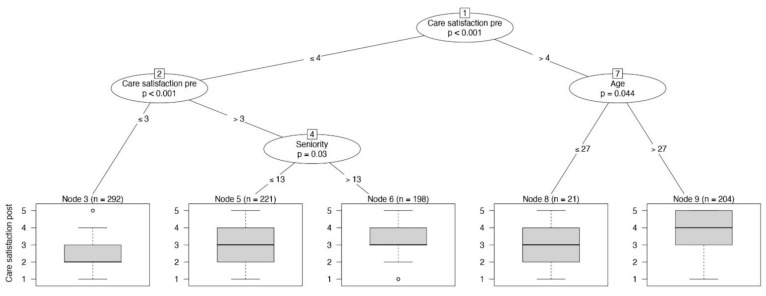
The conditional inference tree with the RNs’ satisfaction with the care provided as dependent variable, reported at the bottom of the figure as boxplot.

RNs’ satisfaction with the care provided remained stable during the pandemic (*p* < 0.001; before: median 4, IQR 3–4; during: median 3, IQR 2–4), but younger nurses (aged ≤27 years) tended to judge their care as poor (*p* = 0.047), with a median Likert scale of 2 (node 12, Figure 6). Similarly, nurses with less work experience (≤13 years) reported a decreased quality of care (*p* = 0.032). RNs who reported high preparedness in terms of education/training showed increased care satisfaction (*p* = 0.039), with a median Likert scale of 4 (node 10, Figure 6).

The results of sensitivity analyses, performed through logistic regressions, confirmed those obtained from the decision trees. In the logistic regression models, the variables associated with the care provided during the COVID-19 pandemic were the same as those identified in the conditional inference trees.

## 4. Discussion

The present study described how nursing care was affected during the first wave of the COVID-19 pandemic and provided indications as to why nurses changed their practices. The first wave of the COVID-19 pandemic hit healthcare systems hard, affecting all aspects of nursing care; more nursing techniques were performed to care for patients, but the nurse–patient relationship suffered. These changes in nursing care led nurses to report lower satisfaction with the care provided.

The impact of the COVID-19 pandemic on nursing care mainly was felt on a clinical level. COVID-19 is a life-threatening illness that requires complex, clinically-focused, and personalized care; many patients were hospitalized for long periods with unpredictable outcomes. The COVID-19 pandemic was influenced by RNs’ previous care habits, indicating the real professional commitment demonstrated by nurses, regardless of the circumstances [22]. Although the pandemic caused high levels of stress among nurses due to the high workload and uncertainty [23], nurses endeavored to guarantee the same level of care they provided before the pandemic to each patient. The prioritization of nursing care provided was affected [24], likely due to the unknown trajectory of the disease, the increase in fundamental care due to high patient dependency, the acquisition of new technical skills, and difficulties in communication and patient education due to infection containment measures.

Nurses who were reassigned to COVID-19 units increased the amount of fundamental care provided. A possible explanation could be that the COVID-19 context often requires many specialized skills, and newly-hired nurses may have preferred to perform more fundamental care while they learned these skills. Conversely, reassigned nurses could have increased the amount of fundamental care simply because COVID-19 patients require more personalized support for their activities of daily living [25]. 

The physiological needs of COVID-19 patients also required the performance of more nursing techniques. In fact, nurses who cared for COVID-19 patients performed these techniques far more frequently during than before the pandemic. Nurses had to adapt to the needs of these patients, often learning or refreshing techniques such as non-invasive ventilation support and arterial puncture for blood gas analysis [26]. Symptom management was also performed more frequently given the higher number of patients as healthcare systems reached capacity [27]. 

A biomedical perspective is often predominant in life-threatening situations, but it should be balanced by the value of caring for others and the individualization of care, something on which technical skills should also focus [28]. This is particularly crucial for COVID-19 patients, who have to face an unknown disease without the support of their loved ones and likely without a close relationship with healthcare providers. Education is the most commonly omitted task when resources are strained [29], and nurse–patient relationships and patient education in our study were reduced due to infection prevention measures [30]. Despite these problems, nurses tried to invent new ways to interact with patients, such as drawing on gowns, printing their pictures to show their faces, and writing their names on face shields [31].

Another relevant finding was the lower satisfaction with care provided among RNs from areas of high COVID-19 prevalence and those who worked in high-intensity units. This may be due to the lack of therapeutic solutions and the patients’ reduced chance of recovery, especially the elderly, making nurses feel less confident that the care they provided was adequate [32]. Nurses working in primary care were also less satisfied with the care provided, probably due to the exceptional number of patients that were treated at home and in other community settings [33].

The devastating, rapid impact that the pandemic has had on the Italian healthcare system might explain some of the lack of preparedness reported by our respondents. During the first wave, COVID-19 was an unknown disease, and a trial and error approach was often applied, with frequent changes in therapies, use of ventilation, and supination [34]; thus, nurses had to adapt their daily work to rapidly changing guidelines and protocols, individualizing the care they provided. Moreover, in Italy, newly-graduated nurses were hired to work in new community care units specifically dedicated to COVID-19 patients, which aimed to identify those who required medical assistance and those who could stay isolated at home with telephone follow-ups [35]. More experienced community care nurses were also recruited for these units, which led to a decreased availability of nurses for established home care services. These factors could have influenced the preparedness of nurses working in community settings. 

The pandemic globally highlighted the nursing shortage and the lack of adequate, coordinated management responses to population health crises [36]. However, contextual factors must be considered when evaluating care outcomes, which should not be merely interpreted quantitatively. In this regard, the patient-to-nurse ratio should not be the only index collected to evaluate staffing needs but, perhaps, environmental and organizational factors should also be considered [37]. Healthcare systems should maintain, and be prepared to implement, plans for pandemic events, and hospital managers should draw up specific emergency plans that define the human resources required in case of a long-term pandemic with recurrent waves, based on competencies, skill mix (e.g., of experienced and novice nurses), and job rotations. Nurse staffing should be proportionate to the clinical complexity of patients and to the organizational complexities to individualize care, minimizing the risk of missed care and patient death [38]. These actions would improve the healthcare system’s response and alleviate the stress imposed by crises, although maintaining the highest standard of nursing care delivered.

The present study has a number of limitations. The majority of the respondents were from the areas most affected by the pandemic in Italy, and most worked in hospital settings. Moreover, our sample could not completely represent the Italian nursing population, which consists of around 367,000 individuals. This may have produced some response bias as nurses from hospital settings may have felt more implicated in the pandemic and, thus, may have been more interested in the survey. Moreover, considering that data collected were self-reported, findings may be affected by the respondents’ emotional or physical condition. High negative and low positive affect have been associated with an emotional autobiographical memory [39]; in the emotionally-charged pandemic, recall may also have influenced our results. Our findings should be interpreted considering that data were collected during the first wave in Italy, the first European country hit by the pandemic, and the first country that had to reorganize its healthcare system to respond to the emergency. Moreover, the use of a cross-sectional design means that causality cannot be proven. However, the decision tree analysis used, and the sensitivity analyses performed, increased the confidence in the inferences.

## 5. Conclusions

This study highlighted the impact of the COVID-19 pandemic on nursing care and the differences between nurses who were and were not reassigned. Despite all of the difficulties faced by nurses, they were generally satisfied with the care provided, except for younger nurses and those with limited working experience. Furthermore, in spite of the healthcare reorganization, and the need to educate, prioritize, and individualize their activities to meet the needs of patients with complex clinical conditions, nurses continued to provide a high level of care, individualizing their practices and ensuring the highest quality of care. Nurses who felt more prepared in terms of education and training were more satisfied with the care provided, and an increase in the number of patients decreased the frequency of fundamental care and nurse–patient relationships. Nurses caring for COVID-19 patients performed nursing techniques more often, to the detriment of patient education. Ensuring the appropriate deployment and education of nurses is crucial to personalize care, especially during a pandemic, and to maintain nurses’ satisfaction with the care provided. Policy makers should consider these results to create structured plans to address long-term pandemics and ensure appropriate nurse staffing in hospitals and primary care settings.

## Figures and Tables

**Table 1:** Participants’ characteristics.

Variables ^1^	Respondents			*p*-Values
	Total Nurses (*n* = 936)	Worked with COVID-19 Patients (*n* = 722, 77.1%)	Worked with Non-COVID-19 Patients (*n* = 214, 22.9%)	
Gender, *n* (%)				
Female	627 (68.2)	474 (66.8)	153 (72.9)	0.011
Male	144 (15.7)	125 (17.6)	19 (9.0)	
Prefer not to say	149 (16.1)	111 (15.6)	38 (18.1)	
Age in years, median (IQR)	39 (30–49)	37 (29–48)	45 (34–51)	<0.001
Educational background, *n* (%)				
Vocational diploma	191 (24.7)	145 (24.2)	46 (26.7)	0.137
Bachelor’s degree	314 (40.7)	257 (42.8)	57 (33.2)	
Professional master’s diploma (1st lev)	177 (22.9)	127 (21.2)	50 (29.1)	
Master’s degree	71 (9.2)	56 (9.3)	15 (8.7)	
Professional master’s diploma (2nd lev)	10 (1.3)	7 (1.2)	3 (1.7)	
PhD	9 (1.2)	8 (1.3)	1 (0.6)	
Geographical area, *n* (%)				
North Italy	560 (72.0)	422 (69.9)	138 (79.3)	<0.001
Lombardy	113 (14.5)	104 (17.2)	9 (5.2)	
Centre and South Italy	105 (13.5)	78 (12.9)	27 (15.5)	
Work experience in years, median (IQR)	14 (5–25)	11 (4–23)	20 (10–29)	<0.001
Working unit, *n* (%)				
High-intensity	296 (39.5)	270 (46.1)	26 (16.0)	0.157
Low-intensity	370 (49.4)	275 (46.9)	95 (58.2)	
Primary care	83 (11.1)	41 (7.0)	42 (25.8)	
Workplace change, *n* (%)				
No	547 (64.1)	387 (58.5)	160 (83.3)	<0.001
Yes	306 (35.9)	274 (41.5)	32 (16.7)	
Preparedness, median (IQR)				
Organizational	2 (1–3)	2 (1–2)	2 (1–2)	0.765
Educational	2 (1–2)	2 (1–2)	2 (1–3)	0.617
Personal	1 (1–2)	1 (1–2)	2 (1–2)	0.073

^1^ Presence of missing data.

**Table 2:** Differences between nurses who changed their work unit.

Variables ^1^	Respondents		*p*-Values
	Reassigned Nurses (*n* = 306, 35.8%)	Not Reassigned Nurses (*n* = 547, 64.2%)	
Gender, *n* (%)			
Female	222 (72.5)	405 (74.0)	0.738
Male	57 (18.6)	87 (15.9)	
Prefer not to say	23 (7.5)	46 (8.4)	
Age in years, median (IQR)	38 (29–48)	40 (30–50)	0.042
Educational background, *n* (%)			
Vocational diploma	67 (21.9)	124 (22.7)	NA
Bachelor’s degree	103 (33.7)	211 (38.6)	
Professional master’s diploma (1st lev)	72 (23.5)	105 (19.2)	
Master’s degree	29 (9.5)	42 (7.7)	
Professional master’s diploma (2nd lev)	4 (1.3)	6 (1.1)	
PhD	4 (1.3)	5 (0.9)	
Geographical area, *n* (%)			
North Italy	189 (61.8)	370 (67.6)	0.003
Lombardy	58 (19.0)	55 (10.1)	
Centre and South Italy	31 (10.1)	72 (13.2)	
Work experience in years, median (IQR)	12 (3.75–24.5)	14 (5–25)	0.057
Number of patients change, *n* (%)			
Decreased	134 (43.8)	255 (46.6)	<0.001
Stable	78 (25.5)	202 (36.9)	
Increased	94 (30.7)	90 (16.5)	

^1^ Presence of missing data; NA Not available.

## Data Availability

The data presented in this study are available on request from the corresponding author. The data are not publicly available due to privacy reason.
